# Bovine Tuberculosis Case Intervention Using the T.SPOT.*TB* Assay to Screen Dairy Workers in Bailey County, Texas

**DOI:** 10.3389/fpubh.2020.00479

**Published:** 2020-09-02

**Authors:** Anabel Rodriguez, David Douphrate, David Gimeno Ruiz de Porras, Emilie Prot, Adriana Perez, Robert Hagevoort, Matthew Nonnenmann

**Affiliations:** ^1^Department of Epidemiology, Human Genetics, and Environmental Sciences, School of Public Health, University of Texas Health Science Center, Houston, TX, United States; ^2^Regional Medical Director, Texas Department of State Health Services, Harlingen, TX, United States; ^3^Department of Biostatistics and Data Science, School of Public Health, University of Texas Health Science Center, Houston, TX, United States; ^4^Department of Agricultural Science Center at Clovis, College of Agricultural, Consumer, and Environmental Sciences, New Mexico State University, Las Cruces, NM, United States; ^5^Department of Occupational and Environmental Health, College of Public Health, The University of Iowa, Iowa City, IA, United States

**Keywords:** bovine tuberculosis (bTB), latent tuberculosis infection (LTBI), Texas, T.SPOT.*TB*, dairy workers

## Abstract

**Background:** One potential exposure on a dairy farm is *Mycobacterium bovis* or bovine tuberculosis (bTB)—an infectious zoonotic pathogen. The prevalence of tuberculosis among dairy workers in the U.S. is unknown largely due to insufficient surveillance and testing practices. Our objective was to determine the prevalence and risk factors of LTBI among dairy workers potentially exposed to cattle infected with bTB in two Bailey County, Texas dairy farms in 2016.

**Methods:** This study involved a secondary analysis of data that were collected by Texas Department of State Health Services (DSHS) Public Health Region 1 (PHR 1). A total of 140 dairy workers were tested using the T.SPOT.*TB* test assay. As a proxy for occupational exposures, we used three categories of cattle exposure groups based on work task, duration, and conditions of exposure to cattle—high, medium, low.

**Results:** Positive LTBI was found among 14/140 (10.0%) of the dairy workers tested with 12/87 (13.8%) in Dairy A and 2/53 (3.8%) in Dairy B. All LTBI cases were determined to be from Hispanic workers with 71.4% indicating having been vaccinated with the BCG vaccine in their country of birth and none indicated previously known exposure to TB. The high category of cattle exposure group experienced the highest prevalence of LTBI (64.3%), followed by the medium cattle exposure group (28.6%), and the low cattle exposure group (7.1%).

**Conclusion:** Our findings suggest that the prevalence of LTBI among dairy workers in Bailey County, Texas is higher than demographically comparable workforces. Future efforts should focus on the development, delivery, and evaluation of a tuberculosis—and other zoonotic diseases—health and safety training which can become a part of a more comprehensive safety management and training program on dairy farms.

## Introduction

Over the last decade (2007–2017), there has been an increase in overall milk production, number of cattle, average herd size per farm, and milk produced per cow in the United States (U.S.) (1, 2). Consequently, these changes in the dairy industry have led to an increase in the demands of labor and the number of employees needed per farm (3). In the U.S., the modern dairy worker is predominantly foreign-born (4). Hispanic male, (5) of ~30 years of age (6) with limited English proficiency and formal education (7). Together, these demographic characteristics translate into a vulnerable workforce (6, 7).

Dairy farm tasks have inherent health and safety hazards which increase the risk for fatal and non-fatal injuries and illnesses among workers (8). One potential exposure on a dairy farm is *Mycobacterium bovis* or bovine tuberculosis (bTB)—an infectious zoonotic pathogen (9–14). *M. bovis* is part of the *M. tuberculosis* complex. Both bTB and TB can cause pulmonary and extra-pulmonary infections, are treated with similar antibiotic regimens, and have indistinguishable confirmative diagnostic tests (15, 16). The true burden of bTB remains underestimated because bTB and TB cases are both clinically classified as solely TB cases (11). In 2018, the U.S. reported a rate of 2.8 TB cases per 100,000 persons—reaching an all-time low (17). Despite diminishing rates, cases of TB remain particularly high among foreign-born individuals residing in the U.S., with 70.2% of reported TB cases originating from foreign-born individuals residing in the U.S (17).

The prevalence of bTB and TB among dairy workers in the U.S. is unknown largely due to insufficient surveillance and testing practices (10, 18). Currently, the Department of Labor's Occupational Safety and Health Administration (DOL-OSHA) does not require producers to test each worker prior to start of employment nor to provide a form of health and safety training on potential transmissible zoonotic diseases, such as bovine TB, on the farm (10). By contrast, the U.S. Department of Agriculture (USDA) and the Food and Drug Administration (FDA) have oversight of enforcement and surveillance of quality control standards on agriculture (19). Bovine TB surveillance among cattle is a common practice for TB accredited-free farm status from the USDA (20). This surveillance inconsistency between cattle and workers becomes a true challenge during epidemiological investigations attempting to establish the etiology of bTB infections on a dairy farm (12, 15, 21). What remains unclear with bovine TB outbreaks is the exact direction(s) of the cross-infection between cattle-to-cattle, cattle-to-person, person-to-cattle, and person-to-person (9).

The primary objective of this investigation is to determine the prevalence and risk factors of latent tuberculosis infection (LTBI) among dairy workers tested using the T.SPOT.*TB* assay on two dairy farms in Bailey County, Texas in 2016. This study was approved by the University of Texas Health Science Center at Houston Committee of the Protection of Human Subjects (CPHS) (HSC-SPH-18-0886) and was given exemption status by the Texas DSHS Institutional Review Board (IRB) in Austin, Texas (IRB# 18-044).

## Materials and Methods

### Study Design

This study involves a secondary analysis of data that were collected by Texas Department of State Health Services (DSHS) Public Health Region 1 (PHR 1) in Lubbock, Texas in response to two requests from the FDA to screen dairy workers potentially exposed to cattle infected with *M. bovis* or bovine tuberculosis (bTB). Dairy workers were employed on two large-herd dairy farms (Dairy A and Dairy B) in Bailey County, Texas (geographically located in the Texas Panhandle) with confirmed cattle bTB active infections. Dairy A and Dairy B are 14.6 miles apart from one another. Texas DSHS PHR 1 personnel conducted primary, field-based data collection among dairy workers on Dairy A and Dairy B. Dairy A had 115 workers employed and Dairy B had 66 workers employed at the time of testing. For this study, a request was made to Texas DSHS to provide de-identified data collected on Dairy A (January 13, 2016; January 15, 2016; April 13, 2016; and April 20, 2016) and Dairy B (July 27, 2016 and October 19, 2016) as well as follow-up data collected on Dairy A (April 20, 2016) and Dairy B (October 19, 2016).

### Study Subjects

The requested dataset contained a total of 140 dairy workers who were interviewed and screened for TB. Subject eligibility included being a male or female worker ≥18 years of age working on both farms with confirmed bTB cattle cases, regardless of job position.

### Data Collection

Texas DSHS bilingual (English and Spanish) personnel visited both farms and administered and logged worker responses to fifty-one questions concerning demographic characteristics, medical history, and previous TB exposure. In addition, certified phlebotomy personnel extracted a 6-mL blood sample required for the T-SPOT.*TB* (Oxford Immunotec, Inc., Marlborough, MA, USA) screening assay at both dairy farm facilities. Public health staff followed-up with both Dairy A and Dairy B in order to test previously unavailable workers and re-test workers with laboratory errors or inconclusive results. Final data and test results were entered into an agency, secured-access, relational database.

### Proxy Exposure

Job position has been proposed as a proxy of occupational exposure to TB for epidemiological investigation concerning bTB on dairy farms (9, 22). For instance, Torres-Gonzalez et al. (14) created three categories of cattle exposure groups based on activity (work tasks), duration, and conditions of exposure to cattle—high, medium, low. High exposure job position was described as workers with direct contact with cattle in confined spaces (e.g., milkers, hospital, maternity, calf-care, supervisors), medium exposure job position was described as workers with direct contact with cattle in non-confined spaces (e.g., breeder, feeder, general worker), and low job position exposure was described as workers with no direct contact with cattle in any type of space (e.g., owners, secretarial staff, ranch/farmers) (14). Work positions collected at the time of the T-SPOT.*TB* test were used to categorized workers appropriately into high, medium, and low exposure groups.

### Data Analyses

Both chi-square and the non-parametric Kruskal–Wallis tests were conducted to explore potential sociodemographic differences between Dairy A and Dairy B. Corresponding *p*-values are shown in Table 1. Summary statistics of demographic characteristics of dairy workers with T-SPOT.*TB* test were reported. Both Fisher's exact test and the non-parametric Kruskal–Wallis tests were conducted to evaluated the association between age, sex, ethnicity, country of birth, category of cattle exposure, and history of BCG vaccine and positive T-SPOT.*TB* test results on Dairy A and Dairy B. Because all positive T-SPOT.*TB* test results in this study were derived from foreign-born dairy workers, statistical analysis resulted in foreign-born being a perfect predictor for a positive T-SPOT.*TB* test result. Therefore, further logistic regression analyses could not be conducted. A type I error level of 0.05 was used to declare significance. Statistical analyses were performed using Stata/SE v.14.0 (23).

**Table 1:** Sociodemographic characteristics of dairy workers TB tested on two Bailey County, TX dairies in 2016 (*n* = 140).

**Characteristics**		**Bailey County, Texas**		
	**Total**	**Dairy A**	**Dairy B**	
	**(*n* = 140)**	**(*n* = 87)**	**(*n* = 53)**	
	**Mean (SD) or** ***n*****(%)**			p^*^
Age	35.5 (12.0)	37.6 (13.5)	32.0 (8.1)	0.0425
Sex				0.2490
Male	126 (90.0)	76 (87.4)	50 (94.3)	
Female	13 (9.3)	10 (11.5)	3 (5.7)	
Ethnicity				0.3440
Hispanic	125 (89.3)	76 (87.4)	49 (92.5)	
Non-Hispanic	15 (10.7)	11 (12.6)	4 (7.6)	
Country of birth				0.0630
United States	21 (15.1)	17 (19.8)	4 (7.6)	
Mexico	82 (59.0)	51 (59.3)	31 (58.5)	
Guatemala	27 (19.4)	15 (17.4)	12 (22.6)	
Honduras	8 (5.8)	2 (2.3)	6 (11.3)	
Recent arrival to U.S. (≤5 years)	43 (30.7)	23 (26.4)	20 (37.7)	0.3460
Travel outside of U.S. past 12 mo.	22 (15.7)	14 (16.1)	8 (15.1)	0.7230
Mexico	19 (86.4)	14 (100.0)	5 (62.5)	
Guatemala	1 (4.5)	0 (0.0)	1 (12.5)	
Honduras	1 (4.5)	0 (0.0)	1 (12.5)	
Philippines	1 (4.5)	0 (0.0)	1 (12.5)	
Years on dairy	3.7 (10.3)	3.1 (4.1)	4.7 (15.9)	0.4237
Work position				0.1080
Milker	47 (34.1)	29 (34.1)	18 (34.0)	
General worker	22 (15.9)	13 (15.3)	9 (17.0)	
Feeder	17 (12.3)	11 (12.9)	6 (11.3)	
Maternity	14 (10.1)	9 (10.6)	5 (9.4)	
Rancher/farmland	11 (8.0)	8 (9.4)	3 (5.7)	
Supervisor/manager	9 (6.5)	7 (8.2)	2 (3.8)	
Breeder	6 (4.4)	1 (1.2)	5 (9.4)	
Hospital	6 (4.4)	1 (1.2)	5 (9.4)	
Calf caretaker	3 (2.2)	3 (3.5)	0 (0.0)	
Secretary	2 (1.5)	2 (2.4)	0 (0.0)	
Owner	1 (0.7)	1 (1.2)	0 (0.0)	
History of working with cattle	65 (46.4)	48 (55.2)	17 (32.1)	0.0280
History of BCG vaccine	81 (57.9)	46 (52.9)	35 (66.0)	0.1260
History of TB treatment	0 (0.0)	0 (0.0)	0 (0.0)	0.4380
History of LTBI treatment	1 (0.7)	0 (0.0)	1 (1.9)	0.2710
History of TB exposure	4 (2.9)	1 (1.2)	3 (5.7)	0.4230
**Other medical history**
Unknown HIV status	27 (19.3)	12 (13.8)	15 (28.3)	0.0350
Immunosuppressive medication	1 (0.7)	1 (1.2)	0 (0.0)	0.4810
Diabetes	4 (2.9)	2 (2.3)	2 (3.8)	0.6110
Leukemia	2 (1.4)	1 (1.2)	1 (1.9)	0.8790
Body weight <10% ideal	4 (2.9)	0 (0.0)	4 (7.6)	0.0140
**History of raw dairy consumption**
Raw milk from dairy	8 (5.7)	4 (4.6)	4 (7.6)	0.4660
Raw milk from dairy taken home	2 (1.4)	1 (1.2)	1 (1.9)	0.3240
Past raw milk consumption	7 (5.0)	2 (2.3)	5 (9.4)	0.1210
Cheese from raw milk	3 (2.1)	2 (2.3)	1 (1.9)	0.4330
Butchered own meat at home	8 (5.7)	7 (8.1)	1 (1.9)	0.0680

* *p-value from X^2^; p-value from Kruskal–Wallis*.

## Results

Table 1 shows the mean age of workers in our sample was 35.5 (SD 12.0) with a range of 18–65 years and 90.0% of participants were male. On the dairies tested, 89.3% of tested workers were Hispanic with 59.0% of participants reporting Mexico as their country of birth, 19.4% Guatemala, 15.1% United States, and 5.8% Honduras. Nearly 31% of workers reported as having recently arrived in the U.S. (within the last 5 years), but only 15.7% had traveled outside the U.S. in the past 12-months, with Mexico (86.4%) being the most common visited destination. On average, tested workers had been employed on their current dairy farm for 3.7 (SD 10.3) years and 46.4% had a history of working with livestock in their country of origin. The majority of workers reported their job position as milkers (34.1%), general workers (15.9%), and feeders (12.3%). Almost 60.0% of participants indicated as having been vaccinated with the bacilli Calmette-Guerin (BCG) vaccine in their country of birth. However, 2.9% indicated history of TB and only one person reported receiving medical treatment for LTBI. In addition, 5.7% reported having a history of consuming raw milk from their dairy of employment and 5.7% had a history of butchering their own meat at home. Statistically significant differences between Dairy A and Dairy B included age, history of working with cattle, unknown HIV-status, and body weight <10% ideal. Compared to Dairy A, Dairy B workers were younger, less experienced with cattle, reported a higher uncertainty of HIV-status, and had lower body fat compositions (<10% ideal).

Table 2 shows the characteristics of dairy workers with positive T-SPOT.*TB* test results. Positive LTBI was found for 10.0% (14 out of 140 workers tested) of dairy workers tested (13.8% of Dairy A workers and 3.8% of Dairy B workers). A follow-up visit was completed for all positive cases. All positive cases tested negative for active TB but were confirmed as LTBI cases. The majority of LTBI cases came from Dairy A (12 out of 14); whereas, Dairy B had two (out of 14) confirmed LTBI cases. All LTBI cases were determined to be Hispanic with 71.4% indicated having been vaccinated with the BCG vaccine in their country of birth and none indicated previously known exposure to TB. Most notable, the high cattle exposure group experienced a LTBI prevalence of 64.3%, followed by the medium cattle exposure group (28.6%), and the low cattle exposure group (7.1%). More specific, one individual with confirmed LTBI had a history of butchering their own meat at home and another confirmed LTBI dairy worker had a history of eating cheese made from raw milk (not shown on Table 2).

**Table 2:** Characteristics of dairy workers with positive T-SPOT.*TB* test results (*n* = 14).

	**Positive T-SPOT.TB test results**			
**Characteristics**	**Total 14 of 140 (10.0%)**	**Dairy A 12 of 87 (13.8%)**	**Dairy B 2 of 53 (3.8%)**	
	**Mean (SD) or** ***n*****(%)**			p^*^
Age	40.4 (13.6)	42.8 (13.2)	26 (1.4)	0.1195
Sex				0.8570
Male	13 (92.9)	11 (91.7)	2 (100.0)	
Female	1 (7.1)	1 (8.3)	0 (0.0)	
**Ethnicity**				
Hispanic	14 (100.0)	12 (100.0)	2 (100.0)	
Country of birth				0.1100
Mexico	9 (64.3)	9 (75.0)	0 (0.0)	
Guatemala	5 (35.7)	3 (25.0)	2 (100.0)	
Category of cattle exposure				0.1100
High	9 (64.3)	9 (75.0)	0 (0.0)	
Medium	4 (28.6)	2 (16.7)	2 (100.0)	
Low	1 (7.1)	1 (8.3)	0 (0.0)	
History of BCG vaccine	10 (71.4)	9 (75.0)	1 (50.0)	0.5050

* *p-value from Fisher's Exact; p-value from Kruskal–Wallis*.

## Discussion

This study found a prevalence of positive LTBI of 10.0% among dairy workers on two dairies under bTB surveillance. These dairy farms represent 20% of dairy farms in Bailey County, Texas in 2016. This is the first case description of an active investigation of bTB on Texas Panhandle dairy farms. In Texas, the total economic impact of dairy products produced and sold is $39.5 billion in addition to the 70,000 direct jobs and 133,000 indirect jobs provided (24)—making it the 3rd largest job generating state, after California and Wisconsin (25). Texas has a total of 400 farms, 5,110 workers, and over 500,000 cattle. Bailey County, Texas has a total of 10 farms, employs over 225 workers, and milks an estimated 22,537 cows (26). Bailey County is an integral part of the Texas-New Mexico milkshed (27).

Previous literature characterized dairy farm workers in Texas and in other dairy states as predominantly an immigrant, (4) Hispanic male, (5) of ~30 years of age (6) with limited English proficiency and formal education (7). However, the prevalence of TB among dairy workers had not been previously established (10). A contact investigation of workers and families on a California dairy farm with a confirmed bTB outbreak reported 43.0% of workers had positive Mantoux tuberculin skin test (TST) results, but no active disease diagnoses with a confirmative chest x-ray follow-up (19).

However, this study reported TB prevalence using the TST, which has a sensitivity of 70%, compared to the sensitivity of the T-SPOT.*TB* test which is 95.6% (17, 28). The challenge with using TST results is that most foreign-born individuals in TB endemic countries are vaccinated as newborn infants with the live-vaccine, BCG; consequently, circulating antibodies cross-react with the tuberculin purified protein derivative (PPD) injected resulting in a false-positive test (29). The T-SPOT.*TB* test is the preferred clinical diagnostic tool of choice for foreign-born and previously BCG-vaccinated individuals, including predominately foreign-born dairy workers (28). This contact investigation was conducted in 2005, 3 years before the FDA commercial approval of the T-SPOT.*TB* assay (28). In addition, 62% of dairy workers and family members reported drinking raw milk from the dairy (19) compared to 5.7% of dairy workers in this study. Also to be noted, this particular study tested dairy workers, family members, and slaughterhouse staff (where 50% of cases came from family members and slaughterhouse workers); whereas, our study only tested dairy workers employed on affected farms (19).

A more recent study conducted by Torres-Gonzalez et al. (14), which used work positions as a proxy for cattle exposure (high, medium, and low cattle exposure) reported a prevalence of 76.2% using the TST and a lower prevalence of 58.5% using an alternative assay to the T-SPOT.*TB* assay. However, this study was conducted in Mexico—a TB endemic country (13, 14, 30). Similar to Torres-Gonzalez et al. (13), our study found that the high cattle exposure group had the highest prevalence of LTBI.

In addition, the prevalence reported in our study (10.0%) was higher than the lifetime TB-prevalence found for U.S. crop-workers between 2000 and 2012 of 0.48% (31). Despite being a demographically comparable workforce, U.S. dairy workers and crop-workers work in different environments and are exposed to different hazards (32). Whereas, crop work is seasonal, dairy production is year round and involves animal handling (33). Modern dairy farms are highly integrated agricultural systems, which consist of numerous work areas involving close interactions with cattle (34). This production system introduces different tasks around the farm with different durations, conditions of exposure to cattle, and routes of bTB exposure (14).

### Intervention Challenges and Study Limitations

Much like the issues faced by dairy farm producers, Texas DSHS experienced similar challenges while following FDA compliance. The FDA contacted Texas DSHS to emergency screen dairy workers at Dairy A and Dairy B. Emergency interventions on dairy farms are uncommon. The first challenge faced was the absence of standard guidelines to test dairy farm workers for bTB. An intake “Dairy TB Evaluation Form” was created using Texas DSHS PHR 1 TB Elimination Program's “TB Initial Health Risk Assessment/History” intake form with the subsequent addition of livestock exposure and raw dairy consumption/meat processing sections. Another challenge faced was the lack of bilingual staff trained on TB interventions. Staff who assisted on this call were chosen only on the basis of speaking both English and Spanish. The majority of staff members had not worked on TB projects nor had ever been trained on TB intervention cases. Due to the emergency nature of this intervention, no trained interpretation services were hired (35).

Study limitations included recall bias of self-reported content collected in the evaluation form such as demographic, exposure risks, raw dairy product consumption, TB symptomology, other medical risks, and previous TB treatment and BCG vaccination. Some workers struggled answering questions and opted to choosing “Unknown” or not answering the question(s) (35). This could have underestimated the history of TB exposure among dairy workers. The majority of dairy workers in the U.S. are of Mexican descent (88.5–97.1%) (6, 7, 36). However, a recent study conducted in New Mexico, Texas, Colorado, Kansas, and New York experienced a large proportion of dairy works of Central American descent, in particular Guatemalan descent (22.7%), and a decreasing percentage of Mexican descent workers (52.4%). The majority of workers identified Spanish as their native language (64.5%); however, 22.4% of workers identified K'iche' (one of 32 Guatemalan languages) as their native language (37). Texas DSHS expressed having a difficult time translating questionnaire and logging answers from the majority of Guatemalan workers (35). Therefore, the unexpected language barrier between staff and K'iche' speaking workers could have led to information bias; and subsequently, differential misclassification of exposures between native English and Spanish speaking workers and native K'iche' speaking workers. Another study limitation is non-response bias. Despite the urgency of the situation, both dairies did not experience a 100% participation rate. Both Dairy A and Dairy B had three working shifts (4:00 A.M, 1:00 P.M, and 8:00 P.M). Due to the remoteness of the dairy locations, Texas DSHS PHR 1 staff missed the first shift of the day (4:00 A.M) (35). In order to make up for this, staff returned to the dairies to conduct follow-up testing and to test workers missing a complete screening. Follow-up also did not experience a 100% participation rate. Currently, the U.S. dairy industry is experiencing significant labor challenges as a result of immigration regulatory policy and differing regional wages and benefits. Consequently, farms are challenged with high worker turnover rates, which complicates any type of follow-up with workers (38, 39).

### Future Plans and Conclusions

Workers should receive a safety training pertaining to transmissible zoonotic diseases—like bovine TB—on a dairy farm. Large animal veterinarians undergo extensive bTB training during professional education (40). Through this training, veterinarians learn the characteristics, transmission, symptoms, diagnostic tests, treatment, and prevention of bTB among cattle. They are also trained on the inherent health hazards while working with bTB suspected cattle and the potential health consequences (41). However, milkers and all other job positions on a dairy farm do not undergo this type extensive professional training education (7, 37). There is a lack of body of literature addressing bTB and TB knowledge among dairy workers. Currently, it is unknown how much dairy workers know about the characteristics, transmission, symptoms, diagnostic tests, treatment, and prevention of TB as well as the potential exposure of bTB on a dairy farm (42). Knowing to what extent dairy workers know and don't know about TB and bTB characteristics, transmission, symptoms, diagnostic tests, treatment, and prevention can help narrow down on the content that needs to be included in a health and safety training pertaining to TB and bTB on a dairy farm. This information can also be used by dairy producers to address training gaps among employed workers. Further development, delivery, and evaluation of TB and bTB health and safety training can be part of a more comprehensive safety management and training program on dairy farms (37).

## Data Availability Statement

The use of the dataset was given exemption status by the Texas DSHS Institutional Review Board (IRB) in Austin, Texas (IRB# 18-044). After the publication of this article, our copy of the dataset will be permanently destroyed. The original dataset is in a Texas DSHS, secured-access, relational database. Requests to access these datasets should be directed to Sandra Morris, SandraA.Morris@dshs.texas.gov.

## Ethics Statement

The studies involving human participants were reviewed and approved by the University of Texas Health Science Center at Houston Committee of the Protection of Human Subjects (CPHS) (HSC-SPH-18-0886) and was given exemption status by the Texas DSHS Institutional Review Board (IRB) in Austin, Texas (IRB# 18-044). The patients/participants provided their written informed consent to participate in this study.

## Author Contributions

AR analyzed and wrote manuscript. DD is lead researcher who helped with manuscript. DG is faculty advisor who helped with data analysis and manuscript. EP is medical director at Texas DSHS who collected data in 2016. AP is lead biostatistician who helped with data analysis. RH is field dairy extension who helped with the manuscript. MN is industrial hygienist who helped with created of manuscript and idea of project. All authors contributed to the article and approved the submitted version.

## Conflict of Interest

The authors declare that the research was conducted in the absence of any commercial or financial relationships that could be construed as a potential conflict of interest.
